# Elevated WBC/HDL Ratio Predicts Poststroke Functional Prognosis

**DOI:** 10.1002/brb3.70995

**Published:** 2025-11-11

**Authors:** Haobo Wang, Xiangqi Kong, Xinyue Yuan, Mina Zhao, Penghong Li, Wei Jing

**Affiliations:** ^1^ Third Hospital of Shanxi Medical University, Shanxi Bethune Hospital Shanxi Academy of Medical Sciences, Tongji Shanxi Hospital Taiyuan China

**Keywords:** inflammation, prognosis, stroke, WBC/HDL ratio

## Abstract

**Background:**

Inflammation and lipid metabolism play critical roles in the pathogenesis and outcome of acute ischemic stroke (AIS). The white blood cell to high‐density lipoprotein cholesterol ratio (WBC/HDL) has been identified as a novel composite biomarker reflecting systemic inflammatory status and anti‐inflammatory capacity. We sought to evaluate the prognostic value of WBC/HDL in predicting 90‐day functional outcomes among AIS patients.

**Methods:**

We retrospectively enrolled 837 AIS patients. Baseline WBC/HDL ratios were calculated at admission. The primary endpoint was 90‐day functional outcome, assessed by the modified Rankin Scale, with scores > 2 defined as unfavorable outcomes. Associations between WBC/HDL and outcomes were assessed through logistic regression, restricted cubic spline (RCS) curves, and subgroup analysis.

**Results:**

Higher WBC/HDL ratios were significantly associated with poor functional outcomes. The association remained robust after adjustment for key covariates (adjusted OR: 1.2589; *p* = 0.0001). The highest WBC/HDL tertile had nearly twice the risk of poor outcomes compared with the lowest tertile. The RCS analysis demonstrated a linear dose–response relationship. Subgroup analyses revealed a consistent relationship across sex, stroke severity, and comorbidity groups, without significant interactions.

**Conclusions:**

The WBC/HDL ratio independently predicts 90‐day functional outcomes in AIS and represents a practical biomarker for early risk stratification.

## Graphical abstract

Elevated admission white blood cell to high‐density lipoprotein cholesterol ratio (WBC/HDL) independently predicts poor 90‐day functional outcomes in acute ischemic stroke patients, supporting its use as a simple biomarker for early risk stratification.

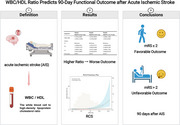

## 1 Introduction

Stroke, a serious cerebrovascular disorder, is a major contributor to global mortality and long‐term disability (Feigin et al. 2018). According to the Global Burden of Disease (GBD) Study 2021, stroke ranks as the third leading cause of death and the fourth leading cause of disability globally, imposing a substantial burden on individuals and healthcare systems alike (GBD 2021 Stroke Risk Factor Collaborators 2024). Acute ischemic stroke (AIS), which constitutes more than 80% of all stroke cases, represents the most common subtype (Saini et al. 2021). Because of its abrupt onset and rapid progression, AIS often results in significant neurological deficits and high rates of disability. Therefore, reliable early prognostic markers are essential to guide clinical decision‐making, tailor individualized treatment plans, and improve patient outcomes.

Mounting evidence indicates that both inflammation and lipid dysregulation are critically involved in the pathogenesis and progression of AIS (Jayaraj et al. 2019; Adibhatla and Hatcher 2008; Etuze et al. 2025). Leukocytes, key components of the innate immune response, are rapidly activated after cerebral ischemia and contribute to blood–brain barrier disruption, oxidative stress, and thrombus formation (Pluta et al. 2021). Increased peripheral white blood cell (WBC) counts, especially neutrophil (Neu) predominance, have been consistently associated with higher stroke severity and poorer clinical outcomes (Jickling et al. 2015, Petrone et al. 2019; Buck et al. 2008; Fang et al. 2017; Maestrini et al. 2015; Shi et al. 2018). In contrast, high‐density lipoprotein cholesterol (HDL) exerts protective effects through its anti‐inflammatory, antioxidant, and endothelial‐stabilizing properties (Tran‐Dinh et al. 2013). Lower HDL levels are linked to an increased risk of cardiovascular events and unfavorable stroke prognosis (Lapergue et al. 2010).

The WBC‐to‐HDL ratio (WBC/HDL) has recently been proposed as a novel composite inflammatory marker that reflects the balance between systemic inflammation and anti‐inflammatory defense. This ratio has demonstrated potential prognostic value in various diseases, including coronary artery disease (Wu et al. 2021), COVID‐19 (Mohammadshahi et al. 2023), and severe stroke (Zou et al. 2025). However, its prognostic significance in patients with AIS remains unclear and has not been systematically investigated.

This study aimed to assess the relationship between the WBC/HDL ratio at hospital admission and 90‐day functional outcomes in AIS patients. We hypothesized that a higher WBC/HDL ratio would be significantly correlated with an unfavorable prognosis. Our findings may provide a simple, inexpensive, and readily accessible biomarker for early risk stratification in AIS and contribute to more personalized and effective stroke management.

## 2 Materials and Methods

### 2.1 Study Population

This retrospective study enrolled patients diagnosed with AIS in the Neurology Department of Shanxi Bethune Hospital from October 2022 to September 2024. The diagnosis of ischemic stroke was based on World Health Organization (WHO) criteria and confirmed by magnetic resonance imaging (MRI) or computed tomography (CT) during hospitalization (Li, Niu, et al. 2024). Smoking history was defined as a history of at least 5 years of smoking with an average consumption of ≥ 10 cigarettes per day, while alcohol use was defined as consumption for more than 5 years with an average intake of ≥ 50 g per day or ≥ 500 g per week (Li, Niu, et al. 2024). Hypertension (HTN), diabetes mellitus (DM), and coronary heart disease (CHD) were identified based on prior diagnoses or diagnoses made during hospitalization.

Inclusion criteria were as follows: (1) diagnosis of AIS confirmed by CT or MRI within 72 h of symptom onset, in accordance with the 2018 Chinese Guidelines for the Diagnosis and Treatment of AIS; (2) first‐ever stroke with no prior history of severe neurological deficits; (3) age ≥ 18 years; and (4) complete clinical data with informed consent obtained.

**FIGURE 1:**
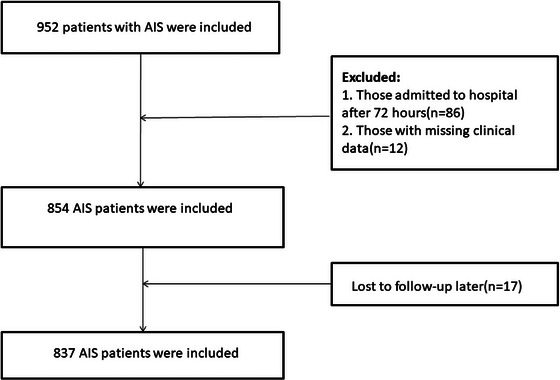
Flowchart of participant selection.

Exclusion criteria included: (1) patients admitted more than 72 h after stroke onset; (2) other vascular ischemic events or cerebral venous thrombosis; (3) intracranial tumors, tumor‐related stroke, or intracranial infections; (4) neurological impairment due to non‐neurological diseases, including malignancy, severe pneumonia, hematologic disorders, or severe cardiac, hepatic, renal dysfunction, or bleeding disorders; (5) prior stroke history with current use of antiplatelet or anticoagulant therapy; (6) patients who had received intravenous thrombolysis or endovascular treatment; (7) chronic inflammatory diseases; (8) incomplete clinical or laboratory data; and (9) loss to follow‐up. Ultimately, a total of 837 eligible patients were included in the final analysis (Figure 1).

### 2.2 Data Collection

Baseline demographic and clinical data were collected, including age, sex, HTN, DM, CHD, smoking, alcohol use, and National Institutes of Health Stroke Scale (NIHSS) score. Venous blood samples (2 mL) were drawn into EDTA‐K2 anticoagulant tubes, and WBC, Neu, monocyte (Mon), lymphocyte (Lym), and platelet counts (PLT) were measured using the Beckman Coulter DxH 800 automated hematology analyzer. Additionally, 5 mL of venous blood was collected in anticoagulant‐free tubes, centrifuged, and analyzed for uric acid (UA), total cholesterol (TC), triglycerides (TG), HDL, low‐density lipoprotein cholesterol (LDL), homocysteine (Hcy), and myeloperoxidase (MPO). D‐dimer was measured using citrated plasma (1:9 sodium citrate) with the ACL‐TOP 750 coagulation analyzer. The WBC/HDL ratio was calculated by dividing total WBC counts by HDL levels.

### 2.3 Outcome Assessment

The primary outcome was functional status at 90 days, assessed using the modified Rankin Scale (mRS). Two trained physicians evaluated the 90‐day outcome via outpatient follow‐up or structured telephone interviews. A favorable outcome was defined as mRS ≤ 2, and an unfavorable outcome as mRS > 2. Stroke severity was also evaluated using the NIHSS score for subsequent subgroup analyses. Mild stroke was defined as NIHSS < 4, while moderate‐to‐severe stroke was defined as NIHSS ≥ 4 (Kwah and Diong 2014).

### 2.4 Feature Selection

**FIGURE 2:**
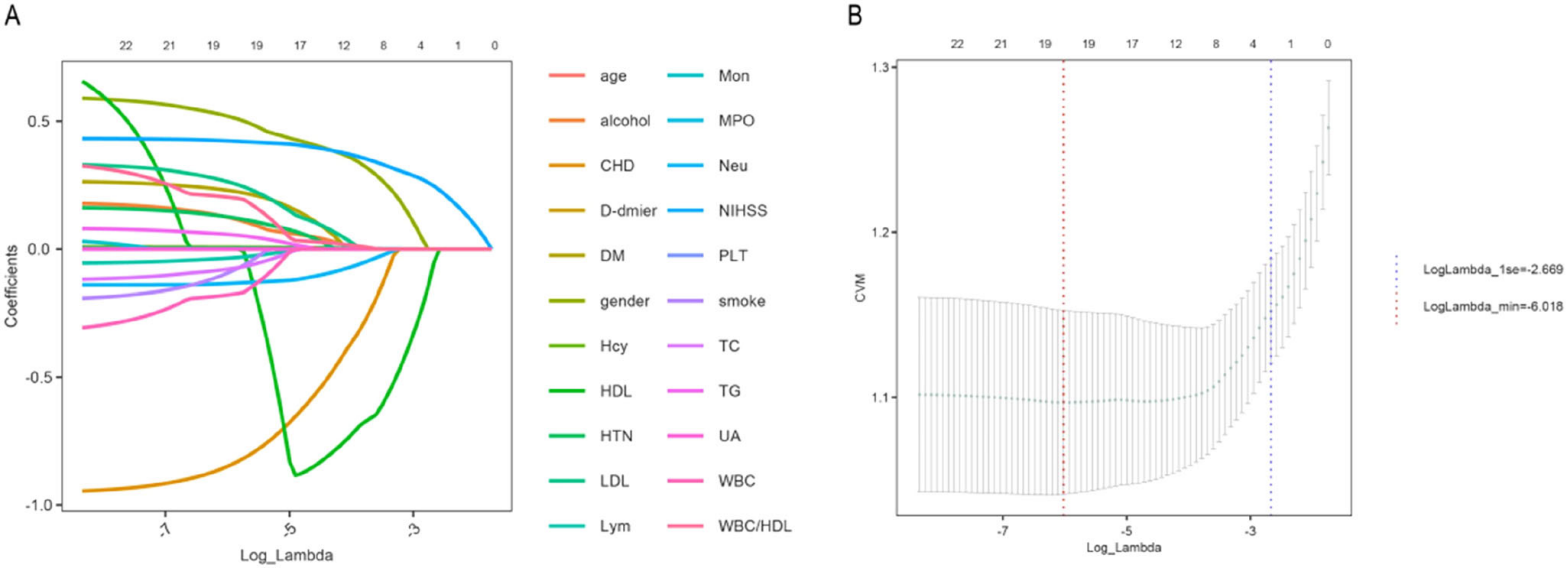
LASSO penalized regression analysis for identifying key variables associated with 90‐day functional outcome of AIS. (A) Coefficient profiles of variables plotted against the log(lambda) sequence. (B) Ten‐fold cross‐validation for tuning parameter selection in the LASSO model. The vertical dashed line indicates the optimal lambda value that minimizes the partial likelihood deviance.

Before evaluating the association between the WBC/HDL ratio and 90‐day outcomes, we performed feature selection using the least absolute shrinkage and selection operator (LASSO) regression (Yue et al. 2025). A total of 18 key predictors were selected: age, sex, NIHSS score, smoking, alcohol use, HTN, DM, CHD, WBC, Lym, Neu, D‐dimer, TG, TC, LDL, MPO, Hcy, and WBC/HDL (Figure 2). These variables were deemed most influential in predicting 90‐day unfavorable outcomes.

### 2.5 Statistical Analysis

Baseline characteristics were compared between patients with favorable and unfavorable 90‐day outcomes. The Kolmogorov–Smirnov test was used to assess the normality of continuous variables, which were reported as mean ± standard deviation or median (with the 25th and 75th percentiles), and compared using the independent *t*‐test or Mann–Whitney *U* test, as appropriate (Mao et al. 2022). Categorical variables were presented as counts and percentages and compared using the chi‐square test.

Multivariable logistic regression was used to examine the association between the WBC/HDL ratio and 90‐day outcomes. Three models were constructed: Model 1 was unadjusted; Model 2 adjusted for demographic variables (age and sex); Model 3 further adjusted for clinical variables, including NIHSS score, smoking, alcohol use, HTN, DM, CHD, WBC, Lym, Neu, D‐dimer, TG, TC, LDL, MPO, and Hcy. Variable selection for multivariable adjustment was informed by clinical relevance, existing literature, and LASSO feature selection.

In sensitivity analyses, the WBC/HDL ratio was categorized into tertiles, and a test for linear trend was conducted to assess robustness. A restricted cubic spline (RCS) regression model with four knots was used to explore potential nonlinear associations between the WBC/HDL ratio and 90‐day outcomes. Subgroup analyses and interaction tests were performed to examine whether the association varied by stroke severity, sex, smoking, alcohol use, HTN, or diabetes status. Each subgroup model was adjusted for the same covariates as Model 3, except for the subgroup variable itself. All statistical analyses were conducted using R version 4.4.2 and DecisionLinc 1.0. Statistical significance was defined as *p* < 0.05.

## 3 Results

### 3.1 Baseline Characteristics of Participants

Among the 837 AIS patients included, 565 (67.5%) achieved favorable 90‐day outcomes, while 272 (32.5%) had unfavorable outcomes (Table 1). The unfavorable outcome group had a higher proportion of males and significantly more current smokers and alcohol users (*p* < 0.05). No significant differences were observed in age or the prevalence of HTN, DM, or CHD (all *p* > 0.05). The NIHSS score was significantly higher in the unfavorable group.

**TABLE 1:** Baselines characteristics of participants.

Characteristic	Overall (*N* = 837)	Favorable outcome (*N* = 565)	Unfavorable outcome (*N* = 272)	*p* value
Gender				< 0.001
Female	259 (31%)	203 (36%)	56 (21%)	
Male	578 (69%)	362 (64%)	216 (79%)	
Smoke				0.002
No	370 (44%)	271 (48%)	99 (36%)	
Yes	467 (56%)	294 (52%)	173 (64%)	
Alcohol				0.008
No	449 (54%)	321 (57%)	128 (47%)	
Yes	388 (46%)	244 (43%)	144 (53%)	
HTN				0.409
No	340 (41%)	235 (42%)	105 (39%)	
Yes	497 (59%)	330 (58%)	167 (61%)	
DM				0.175
No	583 (70%)	402 (71%)	181 (67%)	
Yes	254 (30%)	163 (29%)	91 (33%)	
CHD				0.052
No	765 (91%)	509 (90%)	256 (94%)	
Yes	72 (8.6%)	56 (9.9%)	16 (5.9%)	
NIHSS	2.00 (1.00, 3.00)	2.00 (1.00, 3.00)	3.00 (2.00, 5.00)	< 0.001
Age (years)	63.00 (55.00, 70.00)	62.00 (54.00, 70.00)	63.00 (56.00, 70.00)	0.775
WBC (×10^9^/L)	6.30 (5.30, 7.63)	6.40 (5.30, 7.63)	6.20 (5.30, 7.65)	0.630
Lym (×10^9^/L)	2.12 (1.55, 2.90)	2.11 (1.53, 2.90)	2.18 (1.57, 2.90)	0.621
Mon (×10^9^/L)	0.52 (0.41, 0.63)	0.51 (0.40, 0.64)	0.53 (0.42, 0.62)	0.483
Neu (×10^9^/L)	4.99 ± 2.03	5.06 ± 1.85	4.85 ± 2.35	0.196
PLT (×10^9^/L)	219.00 (182.00, 265.64)	218.08 (182.00, 264.48)	221.50 (183.73, 267.34)	0.596
D‐dimer (ng/mL)	115.00 (70.00, 215.00)	103.00 (67.00, 192.00)	147.50 (85.50, 256.88)	< 0.001
UA (mmol/L)	297.90 (244.40, 376.00)	293.10 (239.80, 365.40)	305.50 (255.30, 386.20)	0.027
TG (mmol/L)	1.44 (1.11, 1.84)	1.44 (1.11, 1.90)	1.45 (1.14, 1.77)	0.986
TC (mmol/L)	4.16 (3.48, 4.88)	4.16 (3.51, 4.88)	4.16 (3.45, 4.84)	0.483
LDL (mmol/L)	2.67 ± 0.75	2.65 ± 0.76	2.70 ± 0.73	0.331
MPO (ng/mL)	138.30 (88.30, 205.80)	121.40 (79.80, 189.60)	171.85 (107.50, 235.55)	< 0.001
HDL (mmol/L)	0.97 (0.85, 1.16)	1.01 (0.88, 1.21)	0.88 (0.79, 1.07)	< 0.001
Hcy (umol/L)	16.20 (12.50, 25.40)	15.70 (12.50, 24.00)	18.05 (13.00, 28.25)	0.004
WBC/HDL	6.35 (5.00, 8.04)	6.21 (4.80, 7.64)	6.94 (5.38, 8.87)	< 0.001

Abbreviations: CHD, coronary heart disease; DM, diabetes mellitus; Hcy, homocysteine; HDL, high‐density lipoprotein; HTN, hypertension; LDL, low‐density lipoprotein; Lym, lymphocyte; Mon, monocyte; MPO, myeloperoxidase; Neu, neutrophil; PLT, platelet counts; TC, total cholesterol; TG, triglycerides; UA, uric acid; WBC/HDL, white blood cell/high‐density lipoprotein; WBC, white blood cell.

Regarding laboratory findings, WBC, Lym, Neu, Mon, and platelets showed no intergroup differences (all *p* > 0.05). However, D‐dimer, UA, MPO, and Hcy levels were significantly elevated in the unfavorable group (*p* < 0.05). Conversely, HDL levels were higher in the favorable group, while TG, TC, and LDL levels did not differ (*p* > 0.05). Notably, the WBC/HDL ratio was significantly increased in the unfavorable group.

### 3.2 Association Between WBC/HDL Ratio and 90‐Day Functional Outcomes

As shown in multivariable analysis (Table 2), the WBC/HDL ratio independently predicts 90‐day functional outcomes. In Model 1, each unit increase in WBC/HDL was associated with a higher risk of poor outcome (OR = 1.1541). This association remained significant after adjustment for age and sex (OR = 1.1373) and persisted in the fully adjusted Model 3 (OR = 1.2589), which accounted for clinical and laboratory covariates.

**TABLE 2:** Multivariable Logistic Regression Analysis of the association Between the WBC/HDL and 90‐day functional prognosis of AIS.

Exposures	Model 1 OR (95% CI), *p* value	Model 2 OR (95% CI), *p* value	Model 3 OR (95% CI), *p* value
WBC/HDL	1.1541 (1.0850, 1.2286), < 0.0001	1.1373 (1.0679, 1.2120), 0.0001	1.2589 (1.1200, 1.4201), 0.0001
Tertiles			
T1	Reference	Reference	Reference
T2	1.2094 (0.8361, 1.7524), 0.3134	1.1033 (0.7577, 1.6084), 0.6082	1.3489 (0.8659, 2.1080), 0.1867
T3	1.9909 (1.3946, 2.8561), 0.0002	1.8157 (1.2640, 2.6188), 0.0013	1.9820 (1.1398, 3.4664), 0.0158
*p* for trend	0.0001	0.0010	0.0159

*Note*: Model 1 =  no covariates were adjusted. Model 2 =  Model 1 + age, gender were adjusted. Model 3 =  Model 2 + NIHSS, smoke, alcohol, HTN, DM, CHD, WBC, Lym, Neu, D‐dmier, TG, TC, LDL, MPO, Hcy were adjusted.

Tertile analysis further supported this relationship. Patients in the highest tertile had nearly double the odds of poor outcome compared with those in the lowest tertile in Model 3 (OR = 1.9820; *p* = 0.0158). A significant linear trend was observed across all models, indicating a dose–response between this ratio and poor outcome risk (*p*‐trend < 0.05).

### 3.3 Evaluation of Nonlinear Association

**FIGURE 3:**
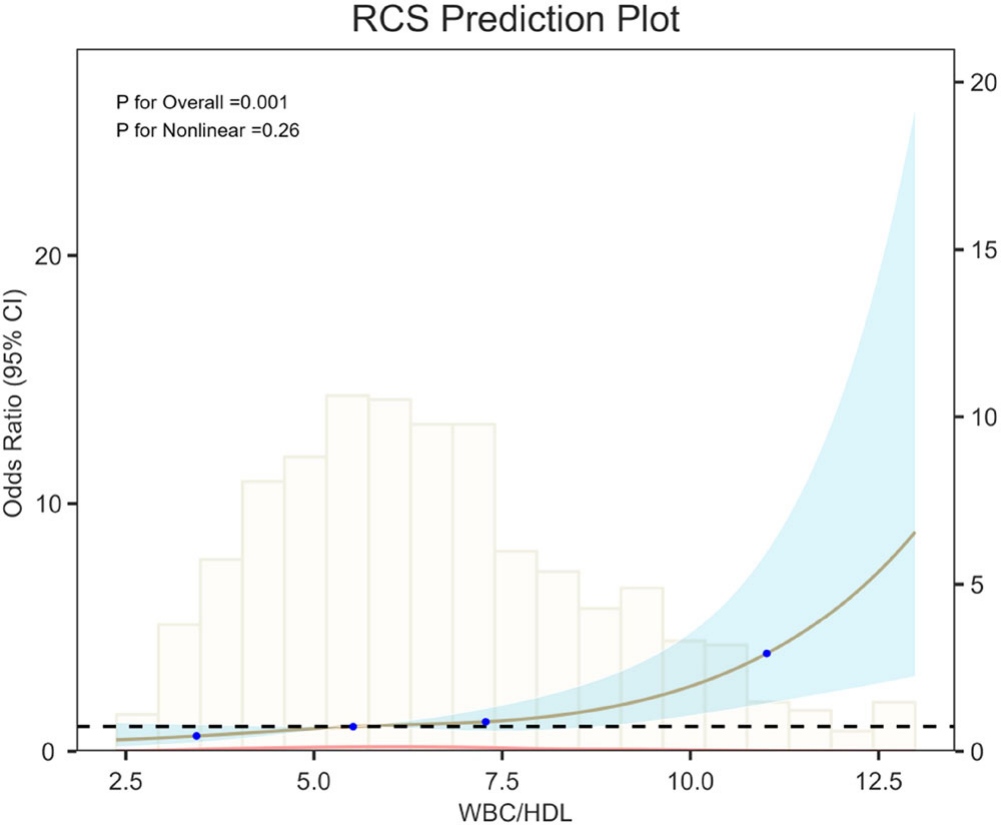
RCS showing the association between WBC/HDL ratio and 90‐day functional outcomes.

RCS analysis (Figure 3) revealed an approximately linear, positive relationship between WBC/HDL and 90‐day unfavorable outcomes (*p* for overall = 0.001). No significant nonlinearity was observed (p for nonlinearity = 0.26), indicating that the risk of poor outcome increased proportionally with higher WBC/HDL levels.

### 3.4 Subgroup Analysis

**FIGURE 4:**
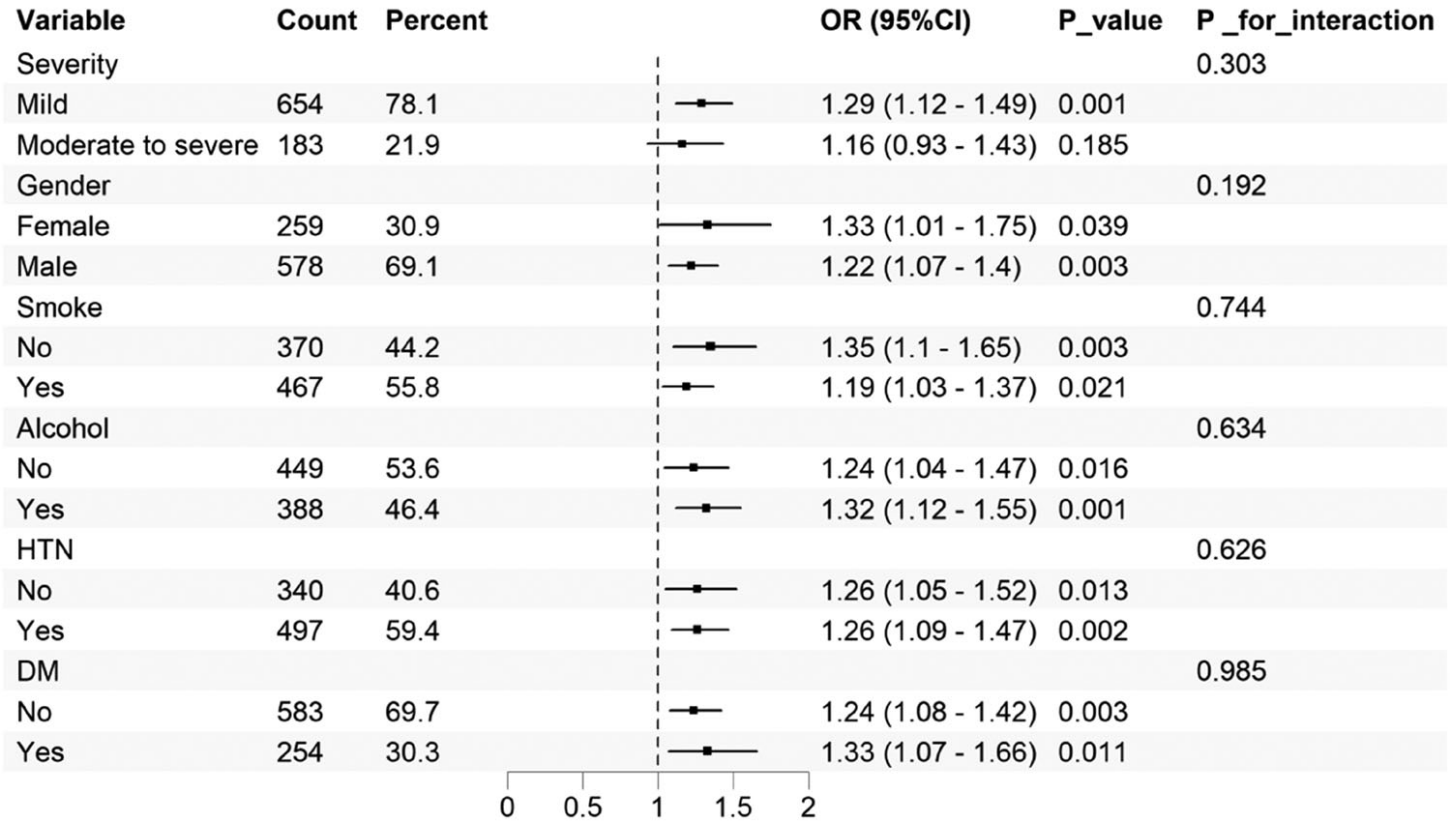
Subgroup analysis for the association between WBC/HDL ratio and 90‐day functional outcomes.

Subgroup analyses (Figure 4) stratified by stroke severity, sex, smoking, alcohol use, HTN, and diabetes consistently demonstrated a positive association between WBC/HDL ratio and unfavorable 90‐day outcomes. No significant interactions were detected, indicating that the prognostic value of this ratio was stable across subgroups (*p* for interaction > 0.05). These findings support this ratio as a robust and generalizable prognostic marker in AIS.

## 4 Discussion

In a large AIS cohort, we systematically evaluated the prognostic value of the WBC/HDL. Our results demonstrated that the WBC/HDL ratio independently predicted 90‐day functional outcomes, even after adjustment for clinical and laboratory covariates. Furthermore, RCS analysis demonstrated a linear dose–response association between this ratio and poor outcomes, and subgroup analyses confirmed the consistency of this association across various clinical populations.

These findings may be interpreted from two complementary perspectives: elevated WBC levels and reduced HDL levels, suggesting a potential synergistic role of neuroinflammation and lipid dysregulation in the prognosis of AIS. Poststroke inflammation is a pivotal contributor to secondary brain injury, and WBCs, as central mediators of the inflammatory cascade, are widely recognized as early indicators of systemic inflammatory activation. Following stroke onset, the release of proinflammatory cytokines and chemokines rapidly recruits WBCs to the ischemic brain regions (Ruhnau et al. 2017). Once activated, WBCs contribute to endothelial injury through the release of proteases, inflammatory mediators, and reactive oxygen species, leading to proteolytic and oxidative damage to endothelial cells, disruption of the blood–brain barrier, thrombogenesis, and secondary neuronal injury (Mazzoni and Schmid‐Schonbein 1996). Moreover, due to their larger size and adhesive properties, WBCs tend to accumulate within the microcirculation, potentially causing microvascular obstruction and the so‐called “no‐reflow” phenomenon, which exacerbates ischemic damage (Ernst 1987; Rasouli et al. 2011). These mechanisms are further supported by clinical studies. Elevated WBC counts in the acute phase of stroke are consistently linked to greater infarct volume, more severe neurological deficits, and poorer functional outcomes at discharge (Buck et al. 2008, Nardi et al. 2012; Hu et al. 2021; Kumar et al. 2013). Elevated WBC counts have been associated with a higher risk of stroke recurrence, highlighting their relevance beyond the acute phase, extending to disease progression and long‐term outcomes (Grau et al. 2004). Recent evidence confirms WBC counts independently predict poor AIS outcomes (recurrence, death, disability) acutely and long‐term (Quan et al. 2019). Therefore, the WBC count may reflect not only the severity of Poststroke inflammation but also act as a key driver of stroke‐related secondary injury.

In contrast to the pro‐inflammatory role of Neu, lipid metabolism—particularly HDL—exerts a protective effect in cerebrovascular events. Beyond its established role in reverse cholesterol transport, HDL demonstrates antioxidant, anti‐inflammatory, antiapoptotic, and endothelium‐stabilizing activities, which underlie its neuroprotective effects in cerebrovascular disorders. At the molecular level, HDL mediates lipid transport across the endothelium via scavenger receptor class B type I (SR‐BI) and ATP‐binding cassette transporters, maintaining vascular lipid homeostasis (Rohrer et al. 2009). More importantly, HDL enhances endothelial function by promoting tight junction integrity, inhibiting apoptosis, reducing glycocalyx shedding, and stimulating proliferation and migration of endothelial cells and progenitor cells, thereby preserving the BBB (Robert et al. 2021). Additionally, HDL activates endothelial nitric oxide synthase to facilitate nitric oxide release for vasodilation and suppresses leukocyte infiltration by downregulating adhesion molecules (Cockerill et al. 1995; Yuhanna et al. 2001). Animal studies have demonstrated that exogenous HDL administration in stroke models mitigates brain edema and preserves BBB integrity (Paternò et al. 2004; Lapergue et al. 2013). Clinical research has further demonstrated that reduced HDL levels are linked to a higher risk, severity, and poor prognosis of stroke (Munshi et al. 2012; Igase et al. 2013; Li, Wang, et al. 2024; Ali et al. 2024). Thus, HDL may be regarded as an endogenous anti‐inflammatory agent, and its depletion reflects a compromised anti‐inflammatory defense.

Taken together, WBC levels reflect the degree of proinflammatory activation, whereas HDL levels represent the effectiveness of anti‐inflammatory defense. The ratio of WBC to HDL may thus offer a more sensitive window into the dynamic immune response following stroke. As a composite indicator of the imbalance between inflammatory burden and anti‐inflammatory capacity, an elevated WBC/HDL ratio may reflect excessive inflammatory activation coupled with insufficient anti‐inflammatory response, ultimately disrupting immune homeostasis and impeding neurological recovery. Compared to individual inflammatory or lipid markers, the WBC/HDL ratio better captures the overall systemic immune‐inflammatory dysregulation in stroke patients. In this study, the observed positive linear association between WBC/HDL and 90‐day functional outcomes further strengthens its theoretical and clinical relevance as a prognostic biomarker. These findings also suggest that WBC/HDL may not only serve as a reliable predictive tool but also constitute a potential therapeutic target for future inflammation‐modulating strategies.

This study extends previous research by providing the first systematic evaluation of the WBC/HDL ratio in association with functional outcomes among a large hospitalized AIS cohort. Prior investigations have explored the prognostic utility of this ratio in risk prediction for various conditions, including coronary artery disease (Wu et al. 2021), COVID‐19 (Mohammadshahi et al. 2023), and severe stroke (Zou et al. 2025). Findings from a MIMIC database‐derived retrospective cohort demonstrated that this ratio was significantly linked to in‐hospital mortality in critically ill stroke patients and outperformed traditional lipid parameters in prognostic accuracy (Zou et al. 2025). However, that study was limited to intensive care unit (ICU) populations and did not account for stroke severity (e.g., NIHSS score), potentially confounding its findings. In contrast, our study validates the prognostic significance of this ratio in a broader AIS inpatient population, with particular emphasis on 90‐day functional outcomes—an outcome of key clinical relevance.

From a clinical perspective, the WBC/HDL ratio offers several advantages: it is derived from routine laboratory tests, is readily available, low‐cost, and simple to calculate, making it feasible for bedside application. In our fully adjusted model, every unit increment in this ratio was linked to a 25.89% elevated risk of adverse outcome. Moreover, the risk of unfavorable outcomes in the highest WBC/HDL tertile was almost double that in the lowest tertile. These results indicate that WBC/HDL could function as a clinically useful and robust marker for early risk stratification in AIS.

Importantly, the linear association observed in the RCS analysis further enhances both the interpretability and clinical utility of this ratio, indicating that the risk of poor outcomes increases progressively with higher ratio values. The absence of significant interactions across subgroups suggests that the predictive value of WBC/HDL is stable regardless of sex, stroke severity, or underlying comorbidities, underscoring its generalizability and robustness as a prognostic biomarker.

Despite providing novel insights, several limitations should be considered. First, due to its retrospective design, causality cannot be established. Second, despite adjusting for numerous confounders, residual confounding from unmeasured factors might still persist. Third, we assessed WBC/HDL levels only at baseline and did not examine their dynamic changes over time, which may also have prognostic implications. Last, as a single‐center study, our findings require external validation in multicenter cohorts to enhance generalizability.

## 5 Conclusion

In conclusion, an elevated WBC/HDL ratio exhibited a significant correlation with unfavorable functional outcomes among individuals with AIS and served as an independent predictor. As a readily available and cost‐effective biomarker, it may aid in early risk stratification and prognostic evaluation among patients with AIS. Additional prospective, multicenter investigations will be essential to confirm its prognostic significance.

## Author Contributions

H.W. and X.K. contributed equally to this work. H.W. was responsible for conceptualization, methodology, formal analysis, and drafting of the manuscript. X.K. contributed to conceptualization, data curation, formal analysis, and drafting of the manuscript. X.Y. participated in investigation, data curation, visualization, and manuscript review and editing. M.Z. contributed to resources, data curation, and manuscript review and editing. P.L. was involved in validation, methodology, and manuscript review and editing. Wei Jing provided supervision, project administration, funding acquisition, and critical review and editing of the manuscript

## Ethics Statement

The study involving human participants was reviewed and approved by the Scientific Research Department of Shanxi Bethune Hospital. Written informed consent was not required for this study.

## Conflicts of Interest

The authors declare no conflicts of interest.

## Data Availability

The data that support the findings of this study are available on request from the corresponding author. The data are not publicly available due to privacy or ethical restrictions.
